# Lactate infusion elevates cardiac output through increased heart rate and decreased vascular resistance: a randomised, blinded, crossover trial in a healthy porcine model

**DOI:** 10.1186/s12967-024-05064-3

**Published:** 2024-03-16

**Authors:** Oskar Kjærgaard Hørsdal, Niels Moeslund, Kristoffer Berg-Hansen, Roni Nielsen, Niels Møller, Hans Eiskjær, Henrik Wiggers, Nigopan Gopalasingam

**Affiliations:** 1 Department of Cardiology, Aarhus University Hospital, Palle Juul-Jensens Boulevard 99, 8200 Aarhus N, Denmark; 2 Department of Clinical Medicine, Aarhus University, Aarhus, Denmark; 3 Department of Heart, Lung, and Vascular Surgery, Aarhus University Hospital, Aarhus, Denmark; 4 Department of Endocrinology and Metabolism, Aarhus University Hospital, Aarhus, Denmark; 5 Department of Cardiology, Gødstrup Hospital, Herning, Denmark

**Keywords:** Lactate, Metabolism, Cardiovascular physiology, Pressure–volume, Haemodynamics, Heart failure

## Abstract

**Background:**

Lactate is traditionally recognized as a by-product of anaerobic metabolism. However, lactate is a preferred oxidative substrate for stressed myocardium. Exogenous lactate infusion increases cardiac output (CO). The exact mechanism underlying this mechanism has yet to be elucidated. The aim of this study was to investigate the cardiovascular mechanisms underlying the acute haemodynamic effects of exogenous lactate infusion in an experimental model of human-sized pigs.

**Methods:**

In this randomised, blinded crossover study in eight 60-kg-pigs, the pigs received infusions with one molar sodium lactate and a control infusion of tonicity matched hypertonic saline in random order. We measured CO and pulmonary pressures using a pulmonary artery catheter. A pressure–volume admittance catheter in the left ventricle was used to measure contractility, afterload, preload and work-related parameters.

**Results:**

Lactate infusion increased circulating lactate levels by 9.9 mmol/L (95% confidence interval (CI) 9.1 to 11.0) and CO by 2.0 L/min (95% CI 1.2 to 2.7). Afterload decreased as arterial elastance fell by  -1.0 mmHg/ml (95% CI  -2.0 to  -0.1) and systemic vascular resistance decreased by  -548 dynes/s/cm^5^ (95% CI  -261 to  -835). Mixed venous saturation increased by 11 percentage points (95% CI 6 to 16), whereas ejection fraction increased by 16.0 percentage points (95% CI 1.1 to 32.0) and heart rate by 21 bpm (95% CI 8 to 33). No significant changes in contractility nor preload were observed.

**Conclusion:**

Lactate infusion increased cardiac output by increasing heart rate and lowering afterload. No differences were observed in left ventricular contractility or preload. Lactate holds potential as a treatment in situations with lowered CO and should be investigated in future clinical studies.

**Graphical Abstract:**

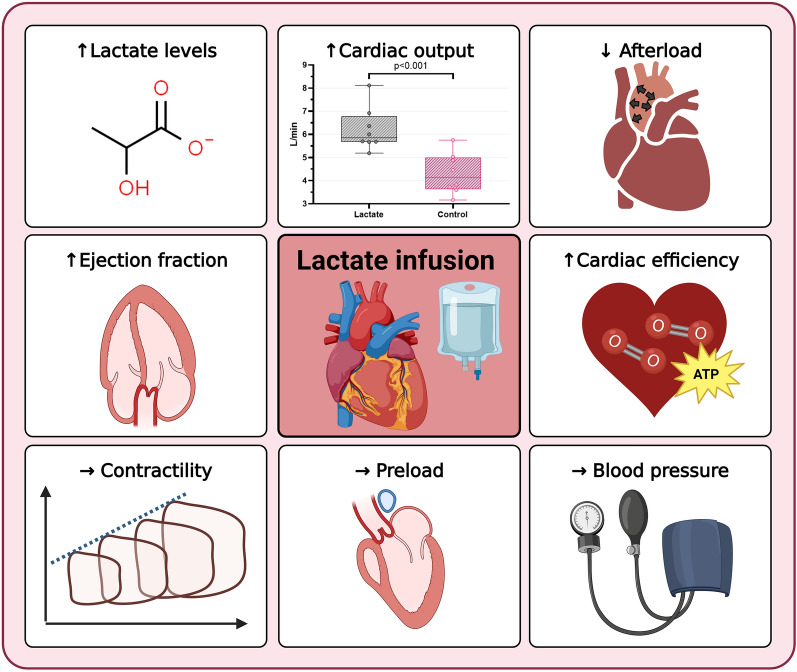

**Supplementary Information:**

The online version contains supplementary material available at 10.1186/s12967-024-05064-3.

## Introduction

The healthy heart is a substrate omnivore that oxidizes fatty acids, carbohydrates, ketone bodies, lactate and amino acids with the preferred substrate being fatty acids [1]. In heart failure (HF), there is a hampered metabolic flexibility characterised by a shift towards metabolic reliance on glycolytic substrates, i.e. ketones and lactate [2–4] along with an upregulation of myocardial transport molecules specific to these substrates [5]. Novel studies have found that substrates such as ketone bodies and lactate may potentially serve as important fuels enhancing cardiac function in HF patients [6, 7].

Increased circulating lactate levels are generally considered a marker of illness severity and have traditionally been acknowledged as a potentially toxic waste product of anaerobic metabolism during hypoxia [8]. Today, however, it is established that lactate production is not confined to anaerobic conditions but occurs in the resting state during aerobic conditions. In fact, lactate is metabolised continuously and immediately by the healthy heart, brain, liver, skeletal muscle, and kidney [9]. Blood lactate levels increase during intense exercise and lactate is commonly used to assess disease severity [10]. However, contrary to conventional beliefs, recent studies have argued that lactate is not the cause of muscle fatigue [11, 12]. Furthermore, lactate is a major contributor to whole-body metabolism and is a readily accessible fuel, even preferred over glucose in some tissues [9, 13, 14]. Additionally, lactate seems to play an important role as a signalling molecule in both intra- and intercellular pathways, bridging glycolysis and oxidative phosphorylation [15].

Lactate infusion increases cardiac output (CO) in patients with acute HF [16], cardiogenic- and septic shock [17], and patients having undergone cardiac surgery [18, 19]. Beneficial hemodynamic and cardiovascular effects of lactate have been found in animal models of endotoxic shock [20–23], haemorrhagic shock [24], acute myocardial infarction [25] and cardiac arrest [26–28]. In healthy volunteers, CO, preload, and mitral annular peak systolic velocity were increased [29]. Until now, the specific mechanisms of action behind these haemodynamic effects of lactate have not been fully elucidated. The aim of this study was to explore the cardiovascular mechanisms underlying the acute haemodynamic effects of lactate infusion in an experimental model of human-sized pigs.

## Materials and methods

### Study design

**Fig.1:**
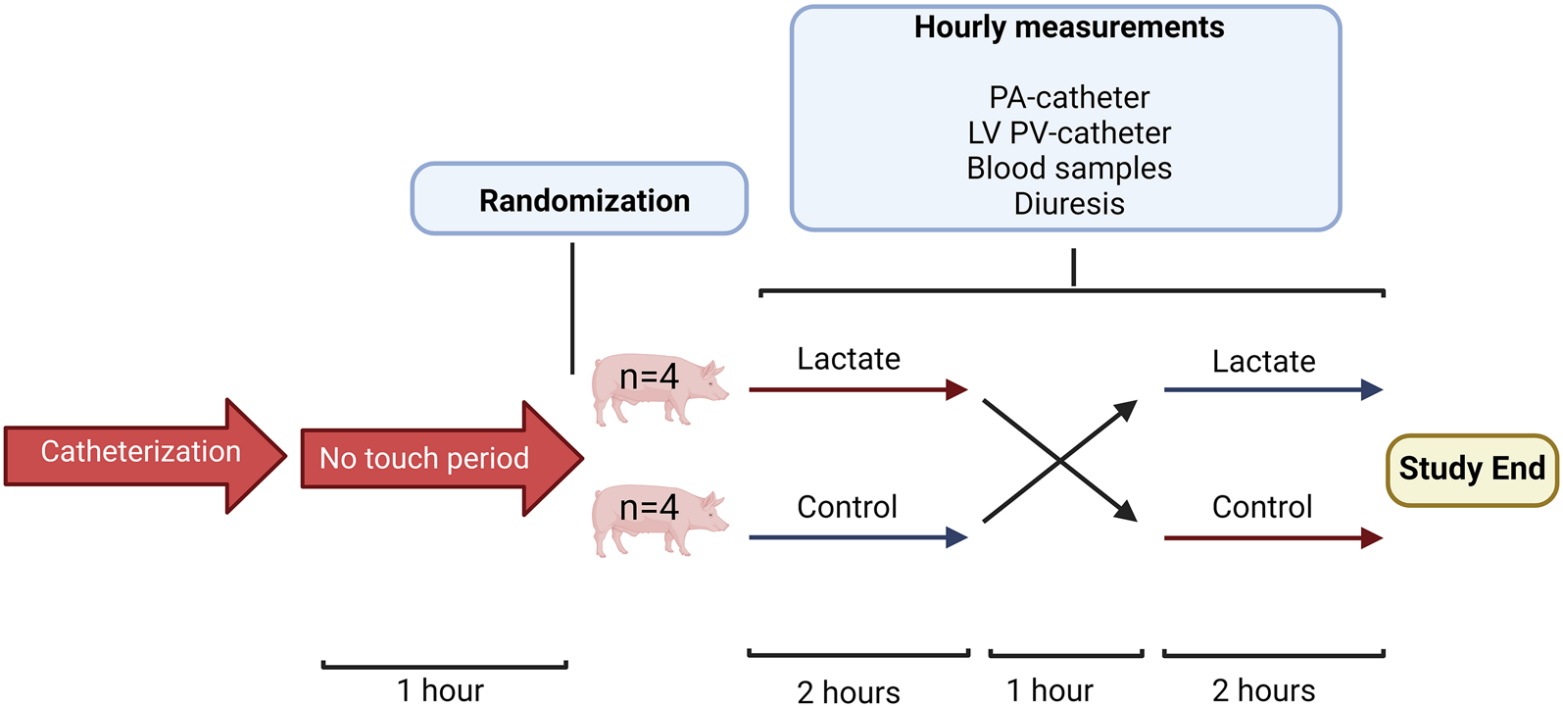
Study design. During instrumentation, the pigs received 1 L of isotonic glucose before a 1-h no-touch period. The study period started at T0, and the pigs were randomised to a treatment sequence group (lactate before crossover to control or control before crossover to lactate, n = 4 in each). The pigs underwent two intervention periods of 2 h separated by a 1-h washout period. During the study periods, hourly measurements were performed as described. *PA *pulmonary artery, *LV PV *left ventricle pressure–volume catheter

The study was a randomised, assessor-blinded, crossover study (Fig. 1). Eight 60-kg female Danish Landrace pigs were randomised into two groups (n = 4 per group) using computer-generated randomisation. Randomisation was performed after instrumentation. Each pig received 5 ml/kg/h molar sodium lactate for two hours and 5 ml/kg/h hypertonic saline for two hours in random order. The two infusion periods were separated by a one-hour washout period with 5 ml/kg/h isotonic sodium chloride (Natriumklorid “B. Braun”, B. Braun Medical, Denmark). During the first hour of anaesthesia, the pigs received 1 L of isotonic glucose (a total of 55 g glucose-monohydrate) to prevent hypoglycaemia. Before baseline measurements, the pigs were allowed a one-hour no-touch period to stabilise while receiving isotonic saline infusion. The study period for each pig was five hours. Investigators were blinded to the intervention sequences. The pigs were continuously monitored and measurements were performed every hour, starting at baseline (Fig. 1). The primary endpoint was change in CO during 120 min of lactate infusion compared with 120 min of control infusion. All secondary endpoints were compared similarly.

### Infusion preparation

The one molar sodium-lactate infusion was made by mixing a two molar sodium lactate solution (SODIO LATTATO 2 mEq/ML, Monico, Venice, Italy) with isotonic saline (Natriumklorid “B. Braun”, B. Braun Medical, Denmark) at a 1:1 ratio producing a solution with 1 mol/L sodium lactate (24.7 g/L of sodium). The control solution was a hypertonic sodium chloride solution with the same amounts of sodium as the intervention solution (24.7 g/L). We chose a hypertonic saline infusion which was matched by osmolality and volume with the lactate infusion to prevent any difference in plasma osmolarity during the infusions, as this is known to cause hemodynamic effects [30–32]. During the one-hour no-touch period and during the one-hour washout period separating the two infusion periods pigs received isotonic saline (9 g/L).

### Anaesthetic management and ventilation protocol

This study enrolled eight 60-kg female Danish Landrace pigs. Prior to transportation to the laboratory facility the pigs were sedated on the farm by intramuscular injection of a commonly used veterinarian anaesthetic mix (Zoletil 50 Vet, Virbac, Denmark) to minimise animal stress and increase refinement. The pigs were intubated immediately upon arrival in the laboratory facility and were admitted to positive-pressure ventilation. Anaesthesia was maintained with continuous intravenous infusion of propofol (3.5 mg/kg/h) and fentanyl (15 µg/kg/h). Anaesthetic adequacy was monitored by testing nociceptive withdrawal and corneal reflexes. The animals were ventilated with a tidal volume of approximately 8 ml/kg and a respiratory rate adjusted to end tidal CO_2_ between 4.5 kPa and 5.5 kPa. Positive end-expiratory pressure (PEEP) was 5.0 cmH_2_O. Body core temperature was measured using a pulmonary artery (PA) temperature probe and we aimed to keep it within the reference range of domestic pigs (38.5 °C to 39.5 °C). A detailed description of animal ethics can be found in Additional file 1.

### Catheterisation

**Fig.2:**
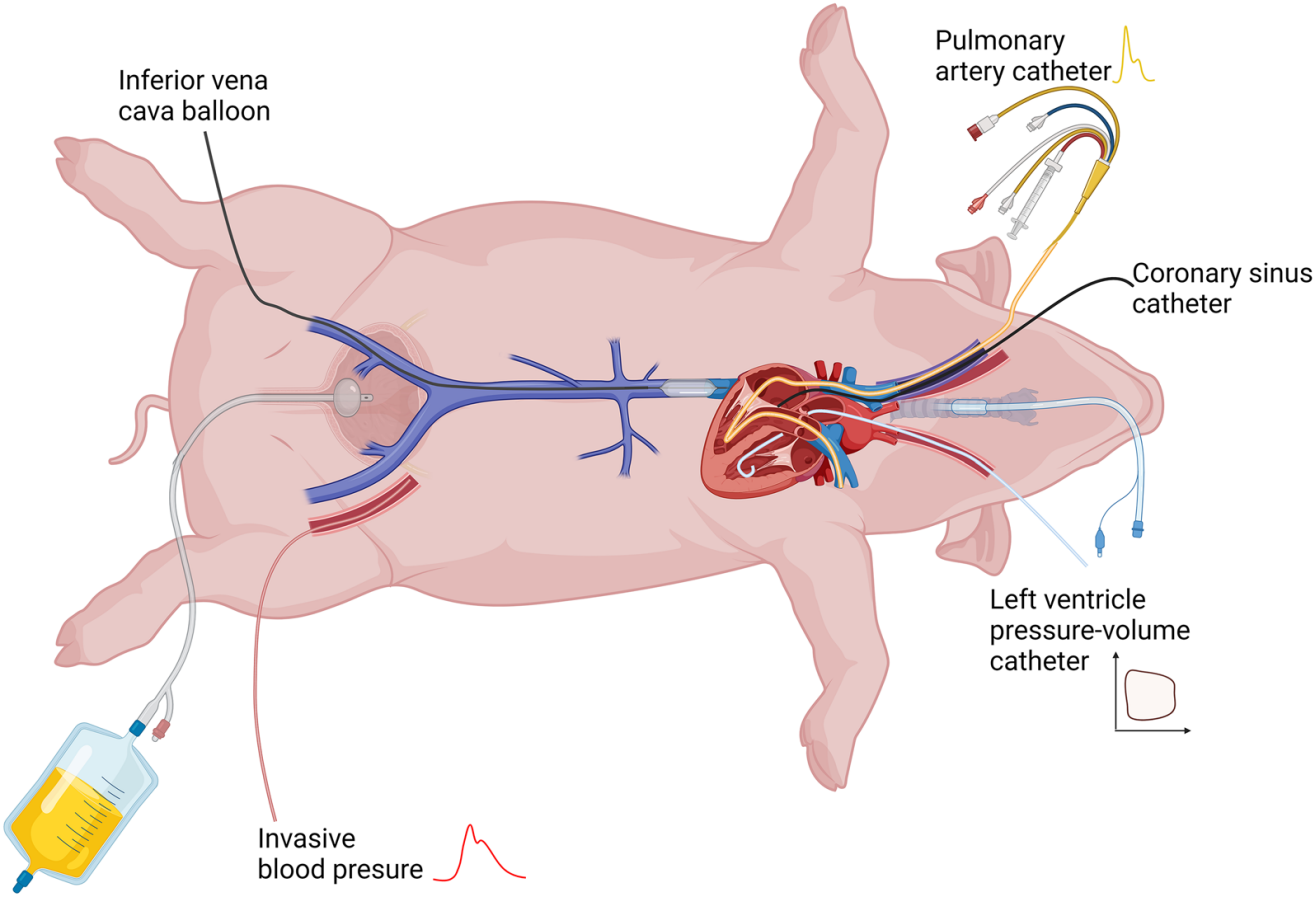
Pig instrumentation. The pigs were intubated and admitted to a ventilator. A Foley catheter was inserted into the bladder before invasive instrumentation. A pulmonary artery catheter and a CS catheter weree placed through the right jugular vein. A PV admittance catheter was inserted in the LV through the left common carotid artery. An occluding balloon was placed in the inferior vena cava at the diaphragm level. Invasive arterial blood pressure and arterial blood samples were sampled from the left femoral artery. *CS *coronary sinus, *LV* left ventricle, *PV *pressure–volume

Invasive catheterisations were performed as shown in Fig. 2. If the pigs developed arrythmias during instrumentation, direct current (DC) conversion was applied.

#### Pulmonary artery catheterisation

A PA catheter was placed through the internal jugular vein under pressure guidance. Correct placement was confirmed by fluoroscopy. CO was measured with the transpulmonary thermodilution method, using a Vigilance box and averaged over three consecutive measurements. A variety of secondary endpoints were assessed with the PA catheter. These endpoints included right atrial pressure (RAP), mean PA pressure (mPAP), PA wedge pressure (PAWP) and mixed venous saturation (SvO_2_), which were all measured hourly. Heart rate (HR) was monitored, and stroke volume (SV) was calculated using data from the PA catheter (SV = CO/HR).

#### Pressure–volume measurements

A pressure–volume admittance catheter was inserted into the left ventricle (LV) through the carotid artery using fluoroscopy. The catheter was fixed and left untouched for the whole study period. A transfemoral occluding balloon was placed in the inferior vena cava at the diaphragm level. The balloon was inflated briefly to perform load-independent contractility measurements of the LV. All measurements were performed hourly during end-expiratory apnoea and recorded using LabChart 8 Pro. Admittance catheters were calibrated according to the manufacturer’s specifications. Before data collection, the system was volume calibrated using a blood resistivity probe and SV calculated from the PA catheter [33]. LV contractility was assessed using end-systolic elastance (Ees) (the slope of the LV end-systolic pressure–volume relationship, LVESPVR) and the maximum rate of pressure generation in the LV (dP/dt_max_). Preload was assessed using end-diastolic volume (LVEDV) and end-diastolic pressure (LVEDP). LV afterload was assessed using arterial elastance (Ea). Additional haemodynamic parameters were assessed, including: stroke work (SW); which is the area inside the pressure–volume loop; potential energy (PE), which is the area on the pressure–volume diagram bounded by LVESPVR, LVEDPVR, and end-systolic portion of the pressure–volume loops; pressure–volume area (PVA), which is the sum of SW and PE representing the total mechanical work of the heart per beat; LV end-systolic volume (LVESV); LV end-systolic pressure (LVESP); LV ejection fraction (LVEF); LV end-diastolic pressure–volume relationship (LVEDPVR) and cardiac efficiency calculated as $\frac{SW}{PVA}$. Data were subsequently analysed assessor blinded in LabChart 8 Pro.

#### Coronary sinus catheterisation

The coronary sinus (CS) was catheterised to calculate the arteriovenous (A-CS) gradient of substrates across the heart. A coronary guiding catheter was placed in the CS through the external jugular vein. The catheter placement was fluoroscopy guided and the catheter was advanced distally to the hemiazygous vein, which, in pigs, connects with the coronary sinus [34]. Correct placement was ensured with a flush of contrast and visualisation of the coronary sinus.

### Other haemodynamic parameters

Mean arterial blood pressure (MAP) was assessed using an intravascular fluid-filled pressure catheter in the femoral artery. The HR was monitored using a three-lead electrocardiogram (ECG). Systemic (SVR) and pulmonary vascular resistance (PVR) were calculated using standard formulae $(SVR=80\times \frac{MAP-RAP}{CO}$ and $PVR=80\times \frac{PAP-PAWP}{CO})$. The veno-arterial CO_2_ tension difference (P(v-a)CO_2_) was calculated $\left({\text{P}}\left({\text{v}}-{\text{a}}\right){{\text{CO}}}_{2}= {Central venous P}_{C{O}_{2}}-Arterial {P}_{C{O}_{2}}\right),$ which is a measure for peripheral tissue perfusion [35].

### Biochemistry and fluid balance

Systemic arterial and venous blood samples from the femoral artery and vein as well as CS blood samples were obtained simultaneously at baseline and every hour during the entire study period (Fig. 1). Lactate, glucose, electrolytes and acid–base parameters (pH, PaCO_2_, HCO_3_^−^) were analysed immediately after sampling. All other samples were stored at − 80 °C and analysed in batches. Insulin levels were analysed with a standard porcine insulin kit (*Mercodia Porcine Insuline ELISA, Mercodia, Uppsala, Sweden).* Free fatty acid (FFA) levels were measured with an enzymatic colorimetric method assay kit (*Wako NEFA-HR(2), Wako Chemicals GmbH, Neuss, Germany).* The ketone body 3-hydroxybutyrate (3-OHB) was measured with hydrophilic interaction liquid chromatography-tandem mass spectrometry (HILIC-MS/MS) with a lower sensitive limit of 5 µmol/L. Apparent strong ion difference (SID) was calculated with a standard formula ((Na^+^  + K^+^  + Ca^2+^  + Mg^2+^)) – (Cl^−^ + Lactate^−^)) [36]. Osmolarity was calculated with a standard formula (2.006 × Na^+^  + 1.228 × Urea + 1.387 × Glucose) [37]. Diuresis was measured hourly using a transurethral catheter. Blood oxygen concentrations were calculated using a standard formula (($\frac{\mathrm{Hgb }\times 1.61 \times 1.36 \times \mathrm{ SO}2 }{100}+\frac{{\text{pO}}2}{0.133322 \times 0.0031})$ where Hgb = haemoglobin concentration in mmol/L, SO_2_ = blood oxygen saturation in percentage and pO_2_ = partial pressure of oxygen in kPa).

### Sample size calculation

The standard deviation of CO (primary endpoint), measured using thermodilution in healthy pigs, is 0.5 L/min (unpublished data from our research facility). By enrolling eight pigs, an effect size of 0.7 L/min would be detected with a power of 90% and a two-sided significance level of 5%.

### Statistical methods

Data were analysed for normal distribution with qq plots and histograms. Normally and non-normally distributed variables are presented as mean ± standard deviation (SD) and median (interquartile range (IQR)), respectively. Continuous data were analysed using a linear mixed effects model with repeated measures to compare the effect of the intervention with the control during the 120 min infusion periods. Residuals were tested for normality. Treatment, time, treatment-by-time interaction, period and treatment sequence were defined as fixed effects, whereas animals were selected as random effects. Our primary analysis compared the change in CO throughout the 120 min of lactate infusion with a 120-min control infusion. The effect of the intervention is presented as the mean with 95% CI. Statistical significance was set at a two-tailed p-value < 0.05. Statistical analyses were made in *R* (*Version 4.2.1, Rstudio, PBC)* and graphics in Prism *(Version 8.4.2, GraphPad, San Diego, CA, USA).* The data supporting the findings of this study are available from the corresponding author upon reasonable request.

## Results

The eight animals were randomised into two groups; one group received lactate before crossover to control; the other group received the infusions in the opposite order (Fig. 1). All the pigs survived until the end of the study period. Four pigs (two in each group) developed atrial fibrillation during the catheterisation period and were all successfully converted with DC conversion before the 1-h no-touch period. None of the pigs developed arrythmias during the study period. Baseline characteristics are shown in Table 1.

**Table 1:** Baseline characteristics

	Lactate to Control (n = 4)	Control to Lactate (n = 4)
Weight (kg)	62 (61–62)	61 (60–62)
MAP (mmHg)	86 (77–93)	84 (69–93)
HR (bpm)	57 ± 12	71 ± 18
CO (L/min)	3.8 ± 0.7	3.7 ± 0.6
RAP (mmHg)	7 ± 3	4 ± 3
mPAP (mmHg)	20 ± 6	23 ± 4
PAWP (mmHg)	11 ± 5	7 ± 2
Blood lactate (mmol/L)	0.80 (0.58–1.08)	0.90 (0.75–1.15)

Results are reported as median (IQR) or mean ± SD

*MAP *mean arterial pressure, *HR *heart rate, *CO *cardiac output, *RAP *right atrium pressure, *mPAP *mean pulmonary arterial pressure, *PAWP *pulmonary artery wedge pressure

### Cardiac output and lactate evolution

**Fig.3:**
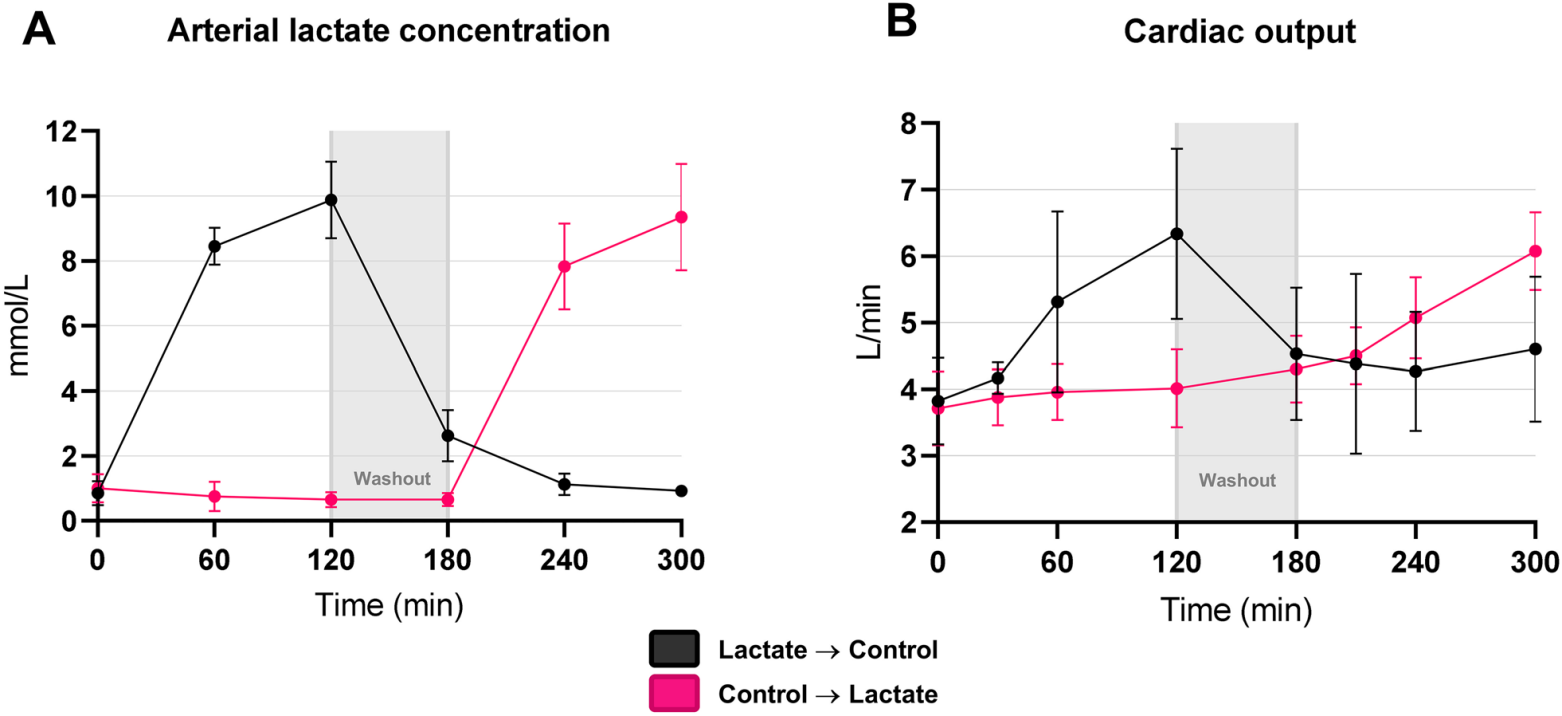
Cardiac output and arterial lactate concentration. Data are expressed as mean ± standard deviation (SD). Temporal differences in lactate levels (**A**) and cardiac output measurements (**B**) plotted from baseline until study end

CO increased by 2.0 L/min (95% CI 1.2 to 2.7, *P* < 0.001) during lactate infusion compared with the control infusion and receded to baseline values during the washout period (Table 2, Figs. 3 and 5, Additional file 1: Fig. S1). Lactate levels increased by 9.9 mmol/L (95% CI 9.1 to 11.0, *P* < 0.001) during lactate infusion compared with control infusion (Table 3, Fig. 3, Additional file 1: Fig. S1). No carryover effect of lactate levels was observed (*P* = 0.20) as the levels returned to baseline values during the washout period.

**Table 2:** Haemodynamic parameters

	Mean after 120 min		Linear mixed model			
	Control	Lactate	Change compared with control	95% CI	*P*-value	*P*-value for interaction
**Pulmonary artery measurements**						
CO (L/min)	4.3 ± 0.9	6.2 ± 0.9	2.0	1.2 to 2.7	** < 0.001**	0.6
HR (bpm)	76 ± 27	88 ± 12	21	8 to 33	**0.003**	0.7
SV (mL)	61 ± 18	72 ± 12	2.4	-13.0 to 18.0	0.8	0.5
SvO_2_ (%)	65 ± 8	73 ± 6	11	6 to 16	** < 0.001**	0.051
P(v-a)CO_2_ (kPa)	1.60 ± 0.59	0.84 ± 0.53	-0.73	-1.10 to -0.35	** < 0.001**	0.4
MAP (mmHg)	87 ± 12	85 ± 15	-3	-8 to 2	0.2	0.7
mPAP (mmHg)	16 ± 3.6	18 ± 3.1	2	-2 to 7	0.4	0.6
RAP (mmHg)	5 ± 3	5 ± 2	-2	-4 to 1	0.2	0.2
PAWP (mmHg)	7 ± 3	8 ± 2	-1	-4 to 3	0.8	0.2
SVR (dynes/s/cm^5^)	1562 ± 335	1042 ± 208	-548	-835 to -261	** < 0.001**	> 0.9
PVR (dynes/s/cm^5^)	189 ± 114	150 ± 42	4	-111 to 126	> 0.9	0.2
**Pressure–volume loop measurements**						
Ea (mmHg/mL)	3.0 ± 1.8	1.9 ± 0.85	-1.0	-2.0 to -0.1	**0.046**	0.5
Ees (mmHg/mL)	0.82 ± 0.27	0.67 ± 0.18	-0.01	-0.31 to 0.29	> 0.9	0.2
LVESV (mL)	150 ± 38	115 ± 27	-51.0	-94.0 to -6.9	**0.032**	0.7
LVEDV (mL)	195 ± 35	173 ± 26	-31	-74 to 12	0.2	0.7
LVESP (mmHg)	108.0 ± 15.0	103.0 ± 21.0	-13.0	-23.0 to -4.2	**0.009**	0.6
LVEDP (mmHg)	16.0 ± 5.6	15.0 ± 3.1	-3.9	-8.7 to 0.8	0.11	0.8
P_max_ (mmHg)	111.0 ± 15.0	109.0 ± 18.0	-9.7	-19.0 to -0.4	**0.050**	0.7
LVEF (%)	25.0 ± 14.0	37.0 ± 13.0	16.0	1.1 to 32.0	0.056	> 0.9
dP/dt_max_ (mmHg × s^−1^)	1315 ± 375	1741 ± 448	502	268 to 737	** < 0.001**	0.2
Tau (ms)	36.0 ± 12.0	39.0 ± 11.0	5.0	-3.7 to 14.0	0.3	0.6
LVEDPVR (mmHg × mL)	0.41 ± 1.10	0.03 ± 0.05	-0.37	-1.10 to 0.34	0.3	0.5
PVA (mmHg × mL)	9312 ± 6843	8589 ± 3997	-2113	-7645 to 3418	0.5	0.3
Potential energy (mmHg × mL)	5464 ± 5087	4405 ± 2408	-3174	-7188 to 841	0.13	0.4
Cardiac efficiency (%)	46 ± 10	51 ± 16	19	6 to 31	**0.008**	0.7
Stroke work (mmHg × mL)	4634 ± 2827	6330 ± 3594	2730	-48 to 5535	0.078	0.6

Mean values are expressed as mean ± SD. Mean values of haemodynamic parameters measured using the PA catheter and the LV PV admittance catheter after 120 min of each infusion. Change compared with control is the development in each outcome during lactate infusion compared with control infusion. **Bold **values indicate *p* < 0.05

*CO *cardiac output, *HR *heart rate, *SV *stroke volume, *SvO*_*2*_ mixed venous saturation, *P(v-a)CO*_*2*_ veno-arterial carbon dioxide difference, *MAP *mean arterial pressure, *mPAP *mean pulmonary artery pressure, *RAP *right atrium pressure, *PAWP *pulmonary artery wedge pressure, *SVR *systemic vascular resistance, *PVR *pulmonary vascular resistance, *Ea *arterial elastance, Ees end systolic elastance (the slope of the end systolic pressure–volume relationship [ESPVR]), *ESV *end systolic volume, *EDV *end diastolic volume, *ESP *end systolic pressure, *EDP *end diastolic pressure, *Pmax *maximum pressure, *EF *ejection fraction, *dP/dt*_*max*_ peak dP/dt, *EDPVR *end diastolic pressure–volume relationship, *PVA *pressure–volume area

**Table 3:** Biochemical parameters

	Mean after 120 min		Linear mixed model			
	Control	Lactate	Change compared with control	95% CI	*P*-value	*P*-value for interaction
pH	7.50 ± 0.11	7.60 ± 0.06	0.22	0.20 to 0.24	** < 0.001**	**0.007**
HCO_3_^−^ (mmol/L)	36 ± 9	47 ± 5	21	19 to 22	** < 0.001**	** < 0.001**
Potassium (mmol/L)	3.7 ± 0.49	3.6 ± 0.69	-0.97	-1.30 to -0.65	** < 0.001**	0.15
Natrium (mmol/L)	160 ± 6	158 ± 8	-3.6	-4.8 to -2.4	** < 0.001**	0.4
Strong ion difference (mmol/L)	49.0 ± 8.2	59.0 ± 2.6	19.0	17.0 to 21.0	** < 0.001**	** < 0.001**
Hgb (mmol/L)	5.90 ± 0.90	5.90 ± 0.42	0.10	-0.22 to 0.42	0.5	0.5
Glucose (mmol/L)	5.20 ± 1.30	6.20 ± 1.00	1.60	0.52 to 2.70	**0.007**	0.4
Lactate (mmol/L)	0.79 ± 0.24	9.60 ± 1.40	9.9	9.1 to 11.0	** < 0.001**	0.2
FFA (mmol/L)	0.40 ± 0.25	0.45 ± 0.36	0.12	-0.28 to 0.52	0.6	0.5
Osmolarity (mmol/L)	330 ± 13	326 ± 15	-5.6	-8.2 to -3.0	** < 0.001**	0.6
Insulin (mU/L)	1.0 ± 0.4	3.5 ± 2.0	3.2	1.6 to 4.8	** < 0.001**	> 0.9
3-hydroxybutyrate (µmol/L)	6.6 ± 4.5	5.8 ± 6.0	-1.5	-6.0 to 3.1	0.5	0.9
Diuresis (mL)	138 ± 189	107 ± 75	18	-176 to 212	0.9	0.082

Mean values are expressed as mean ± standard deviation (SD). Mean values of biochemical parameters measured in arterial blood after 120 min of each infusion. Change compared with control is the development in each outcome during lactate infusion compared with control infusion. **Bold **values indicate *p* < 0.05

*HCO3−*  bicarbonate, *Hgb* haemoglobin, *FFA * free fatty acids

### Haemodynamic and oxygenation parameters

SvO_2_ increased by 11 percentage points (95% CI 6 to 16, *P* < 0.001) and P(v-a)CO_2_ decreased by  -0.73 kPa (95% CI -1.10 to  -0.35, *P* < 0.001) during lactate infusion compared with control infusion. HR increased by 21 bpm (95% CI 8 to 33, *P* = 0.003) when comparing the lactate infusion period with the control infusion period, whereas SVR decreased by  -548 dyn-s/cm^5^ (95% CI  -835 to  -261, *P* < 0.001). No significant change in MAP, RAP, mPAP, PAWP, SV or PVR was observed (Table 2 and Fig. 5, Additional file 1: Fig. S2).

### Pressure–volume measurements

**Fig.4:**
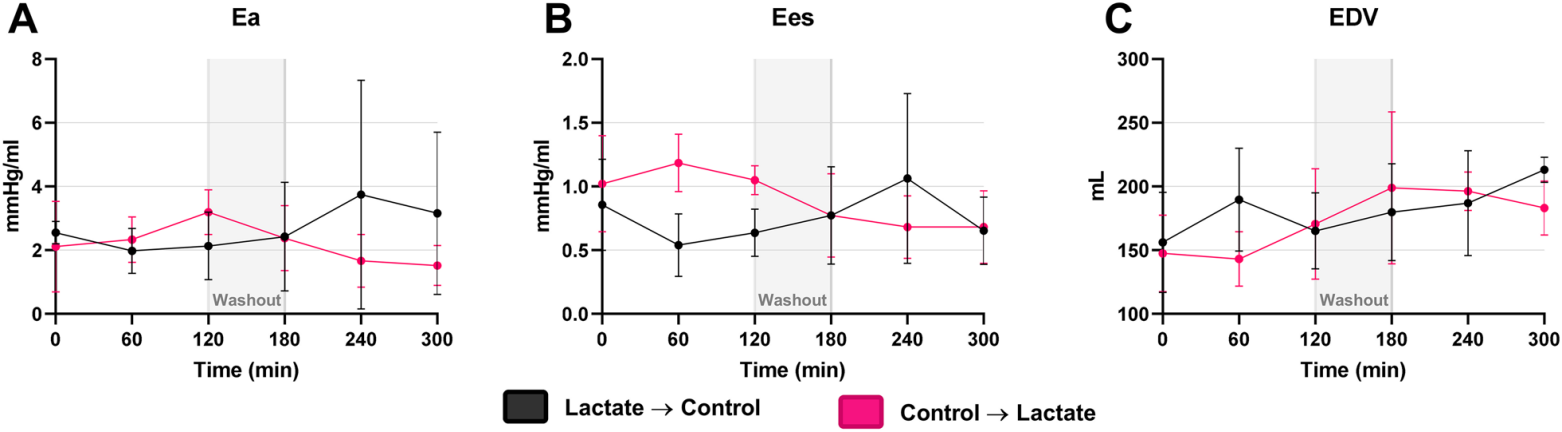
Afterload, contractility and preload. Data are expressed as mean ± standard deviation (SD). Arterial elastance (Ea), end systolic elastance (Ees) and end diastolic volume (EDV) for both groups during the study period shown on graph** A**,** B** and** C**, respectively. The grey masked area marks the washout period

**Fig.5:**
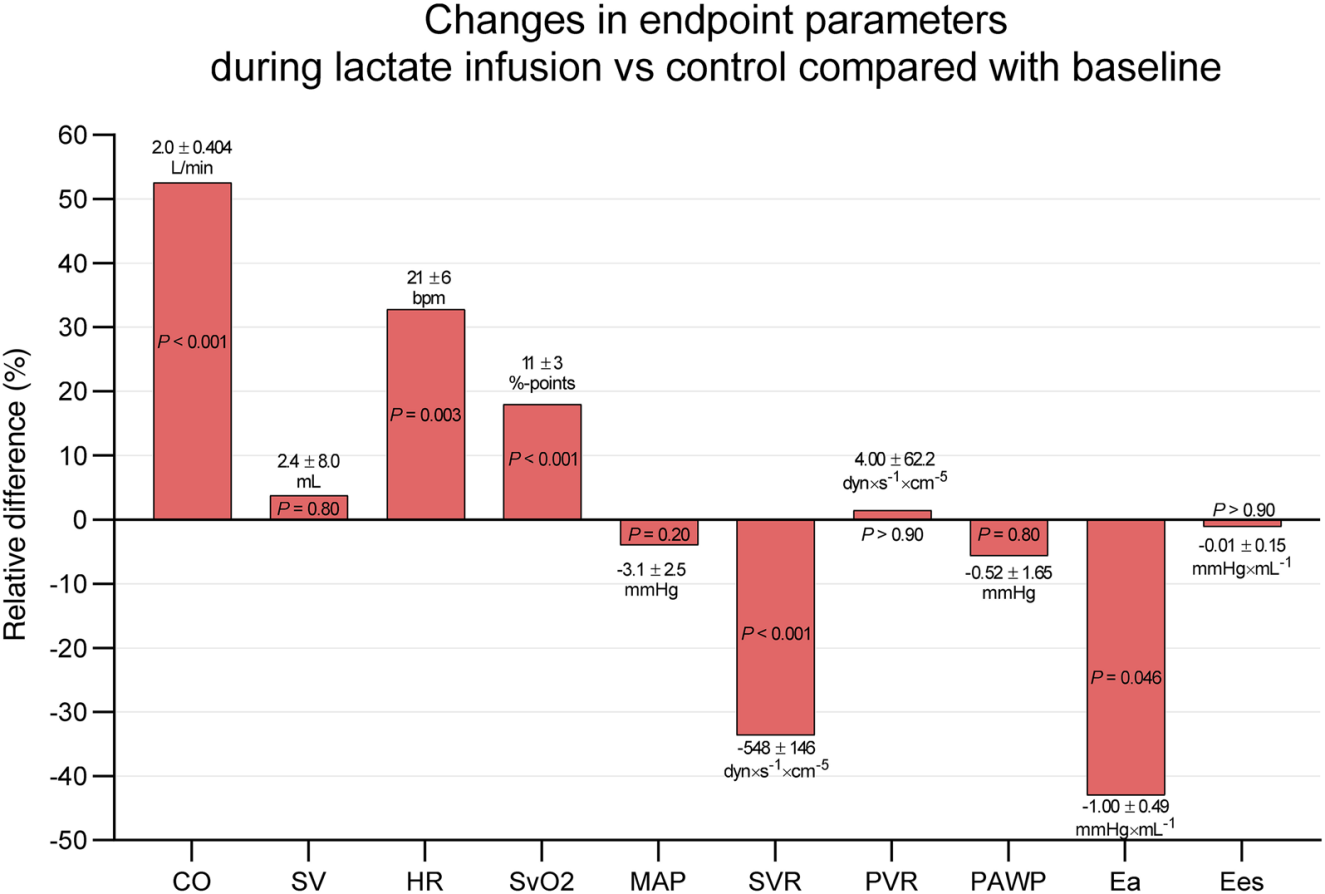
Changes in endpoint parameters during lactate infusion versus control compared with baseline. Mean relative change during lactate infusion compared with control infusion. Corresponding mean absolute changes ± standard error of mean (SEM) are listed above or below each bar. P-values are stated on all results. *CO *cardiac output, *SV *stroke volume, *HR *heart rate, *SvO2 *mixed venous saturation, *MAP *mean arterial blood pressure, *SVR *systemic vascular resistance, *PVR *pulmonary vascular resistance, *PAWP *pulmonary artery wedge pressure, *Ea *arterial elastance, *Ees *end systolic elastance, *bpm *beats per minute

The afterload measurement, Ea, decreased by  -1.0 mmHg/ml (95% CI  -2.0 to  -0.1, *P* = 0.046) during lactate infusion compared with control infusion (Figs. 4A and 5). Another afterload surrogate measurement, LVESP, decreased by -13.0 mmHg (95% CI -23.0 to -4.2, *P* = 0.009). During lactate infusion, peak LV pressure, P_max_, decreased by  -9.7 mmHg (95% CI  -19.0 to  -0.4, *P* = 0.050). LVESV decreased by -51.0 ml (95% CI -6.9 to -94.0, *P* = 0.032). The load-independent contractility measurement, Ees, did not change significantly during lactate infusion ( -0.01 mmHg/ml, 95% CI  -0.31 to 0.29, *P* > 0.90) (Figs. 4B and  5). In contrast another contractility measurement, dependent on contractility velocity, dP/dt_max_, increased by 502 mmHg/s (95% CI 268 to 737, *P* < 0.001) during lactate administration (Table 2). The preload measurements LVEDV and LVEDP did not change significantly during lactate infusion compared with control infusion (Fig. 4C). An increase in LVEF of 16.0 percentage points (95% CI 1.1 to 32.0, *P*  = 0.056) was observed during lactate infusion compared with control infusion. Cardiac efficiency increased by 19 percentage points (95% CI 6 to 31, *P* = 0.008). PVA, PE, or SW of the LV were not significantly different between lactate infusion and control infusion. In the analysis, we found no interaction of the treatment sequence for any of the parameters (Table 2). Additional file 1: Fig. S3 shows a schematic drawing of a mean loop for lactate infusion and control infusion.

### Cardiac extraction of substrates

**Fig.6:**
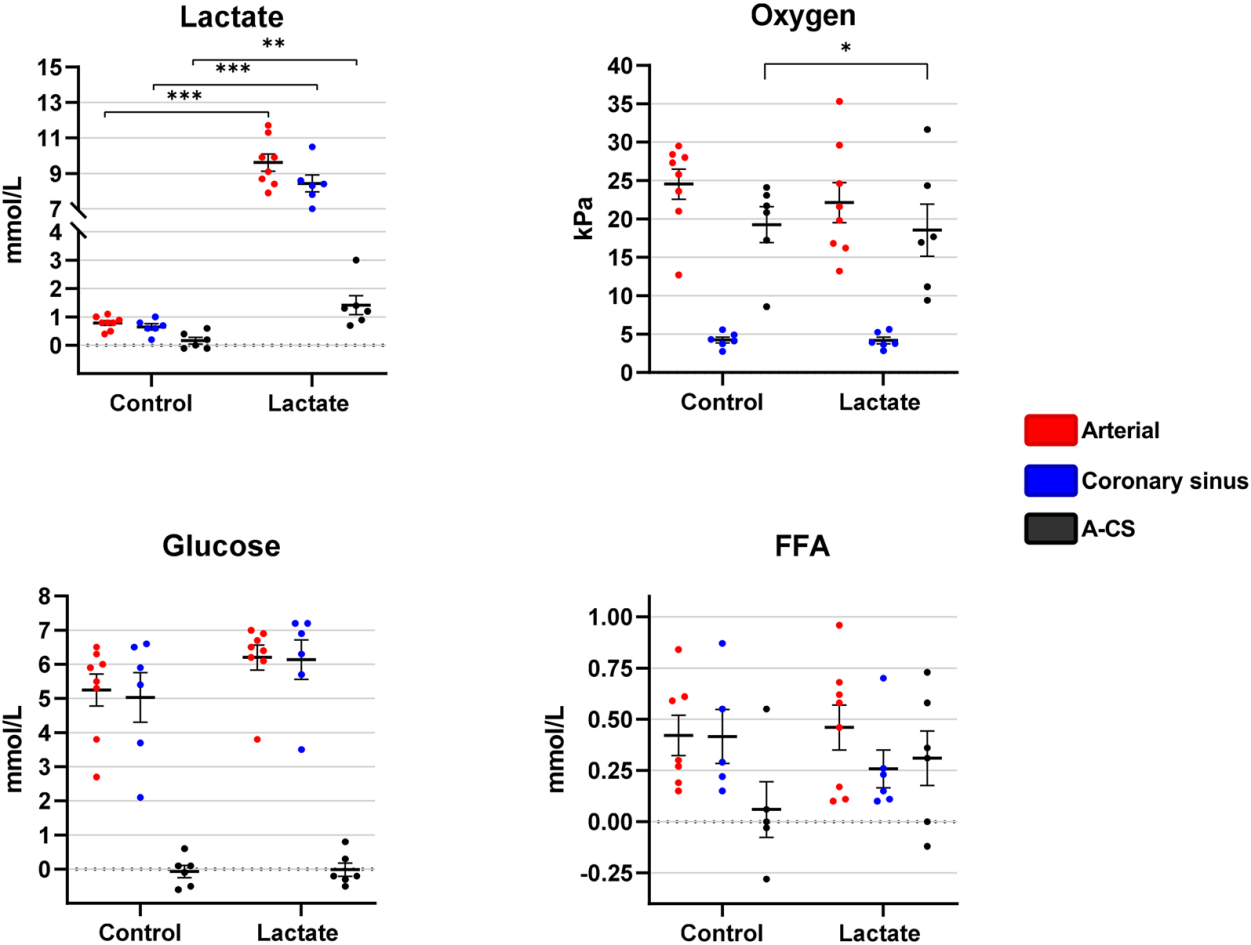
Arteriovenous difference of metabolites across the heart. Data are presented as mean ± standard error of mean (SEM). Mean difference of lactate, oxygen, glucose and free fatty acids across the heart after two hours of control and two hours of lactate infusion, respectively. Red circles: arterial blood samples, blue circles: coronary sinus blood samples, black circles: arterial versus coronary sinus difference. FFA = free fatty acids. Horizontal black lines above data sets mark significant differences. ***indicates *P* < 0.001, **indicates *P* < 0.01, *indicates *P* < 0.05

The A-CS gradient of lactate across the heart increased by 1.20 mmol/L (95% CI 0.56 to 1.90, *P* = 0.002) during lactate infusion compared with control infusion (Table 4, Fig. 6). The A-CS oxygen gradients across the heart decreased by  -1.80 mg/dL (95% CI  -3.30 to  -0.18, *P* = 0.041) during lactate infusion compared with control infusion. No difference was found regarding FFA or glucose levels across the heart. We found no interacting effects of treatment sequence.

**Table 4:** A-CS difference of metabolites across the heart

	Mean after 120 min		Linear mixed model			
	Control	Lactate	Change compared with control	95% CI	*P*-value	*P*-value for interaction
Lactate (mmol/L)	0.17 ± 0.29	1.40 ± 0.82	1.20	0.56 to 1.90	**0.002**	0.4
Glucose (mmol/L)	-0.07 ± 0.44	-0.02 ± 0.48	0.08	-0.46 to 0.63	0.8	0.6
FFA (mmol/L)	0.04 ± 0.34	0.31 ± 0.33	0.42	0.03 to 0.80	0.054	0.7
Oxygen (ml/dL)	7.60 ± 2.20	6.50 ± 2.30	-1.80	-3.30 to -0.18	**0.041**	0.5

Mean values are expressed as mean ± standard deviation (SD). Mean values of the A-CS difference of metabolites and oxygen across the heart after 120 min of each infusion calculated as A-CS difference. Change compared with control is the development in A-CS difference during lactate infusion compared with control infusion. **Bold **value indicate *p* < 0.05

*FFA *free fatty acids

### Biochemical parameters and fluid balance

pH increased by 0.22 (95% CI 0.21 to 0.24, *P* < 0.001) and arterial bicarbonate (HCO_3_^−^) levels increased by 21 mmol/L (95% CI 19 to 22, *P* < 0.001) during lactate infusion compared with control infusion (Table 3 and Additional file 1: Fig. S4). We found a carryover effect (*P* = 0.007 and *P* < 0.001 for pH and HCO_3_^−^ level, respectively). During lactate infusion, potassium levels decreased significantly by -0.97 mmol/L (95% CI -0.65 to -1.30, *P* < 0.001), glucose increased by 1.60 mmol/L (95% CI 0.52 to 2.70, *P* = 0.007) and insulin increased by 3.2 mU/L (95% CI 1.6 to 4.8, *P* < 0.001) as compared with control. Sodium increased during each infusion period (Additional file 1: Fig. S4). However, the increase was greater during control infusion, amounting to 3.6 mmol/L (95% CI 2.4 to 4.8, *P* < 0.001). Osmolarity was also higher during control infusion than during lactate infusion, amounting to 5.6 mmol/L (95% CI 3.0 to 8.2, *P* < 0.001). We found no differences in 3-OHB blood levels during any of the infusion periods. We found no difference in diuresis during lactate infusion compared with control infusion (Table 3).

## Discussion

In this experimental, randomised, blinded study on human-sized pigs, we compared exogenous infusion of one molar sodium lactate and a matched iso-osmolar and iso-volemic sodium chloride control infusion. Lactate infusion increased CO by 2.0 L/min, LVEF by 16 percentage points and HR by 21 bpm. The increase in CO was mediated through a decrease in Ea and SVR (afterload) without any change in MAP. The load-independent left ventricular contractility measurement (Ees) remained unchanged during lactate infusion compared with control infusion as did also LVEDV and LVEDP (preload). Infusion of lactate increased cardiac efficiency, SvO_2_ and A-CS gradients of lactate across the heart and decreased myocardial A-CS oxygen gradients.

### Hemodynamic findings

To our knowledge, the present study is the first to investigate the acute effects of lactate on cardiac haemodynamics using an LV pressure–volume catheter in a large animal model. Thus, the present study provides comprehensive insight into the haemodynamic mechanisms underlying cardiovascular changes during lactate infusion.

The main finding of this study was that CO increased by 2.0 L/min during the two-hour lactate infusion as compared with control infusion. Furthermore, we found that afterload, as measured by Ea and SVR, decreased during lactate infusion as compared with control infusion. This effect, in combination with increased HR, enhanced the CO. In contrast, the systemic blood pressure remained unchanged. Furthermore, we observed a decrease in P(v-a)CO_2_ during lactate infusion. P(v-a)CO_2_ is inversely related to CO and is a surrogate indicator for the adequacy of venous blood flow to wash out CO_2_ produced by peripheral tissues [35]. Thus, the observed decrease in P(v-a)CO_2_ during lactate infusion indicates an improved peripheral perfusion as a result of the increased CO. We found no change in the load-independent contractility measure Ees during lactate infusion. We did find an increase in dP/dt_max_ during lactate infusion compared with control infusion. As we found no changes in preload (LVEDV), the greater dP/dt_max_ was likely due to increased HR despite lower ventricular pressure generation. A rise in HR is associated with a stronger contractile force of the myocardium, a phenomenon known as force-frequency relationship [38]. dP/dt_max_ is defined as the time for LV pressure increase during the initial phase of contraction during ejection. Therefore, the elevated dP/dt_max_ during lactate infusion could likely be explained by the elevated HR’s effect on the force-frequency relationship.

### Cardiac oxygen utilisation

In human patients with congestive HF, increased lactate myocardial lactate consumption improves cardiac efficiency [39]. Other studies have shown that lactate improves cardiac efficiency after haemorrhagic shock in rats [24]. Additionally, endotoxic and haemorrhagic shock rat models have shown that myocardial lactate deprivation impairs cardiac metabolism, leading to compromised myocardial function, poorer outcome and earlier death [22, 40]. Hence, lactate has been suggested as a key metabolite that is readily oxidized in stressed cardiomyocytes [2, 16, 17]. During lactate infusion, we found that the A-CS difference in oxygen levels decreased. Despite no significant difference in PVA, PE or SW, we observed a significant increase in cardiac efficiency from the LV pressure–volume data. The observed improvement in cardiac efficiency may be attributable to a shift in myocardial substrate selection favouring increased reliance on lactate [14, 39]. In healthy state the heart prefers FFAs as oxidative substrate [1]. FFAs have a high ATP production per molecule but have a lower ATP per oxygen molecule yield than other substrates i.e. glucose, ketones, and lactate [41, 42]. The present study found an increased gradient of lactate concentration across the heart. This finding might indicate an increase in myocardial consumption of lactate, even though coronary blood flow was not measured. Also, increased lactate levels have been shown to correlate with increased myocardial uptake of lactate [14]. Hence, an increased myocardial consumption of lactate could explain the increased cardiac efficiency. Importantly, HR was increased during lactate infusion. HR is a determinant of MvO_2_ [43]. Even though PVA, a parameter closely correlated to MvO_2_ during varieties in HR and loading conditions [44] remained unaltered, further studies are needed to further elucidate the mechano-energetic effects of LV during lactate infusion.

### Circulating metabolites

Blood glucose and insulin levels increased during the lactate infusion period (Table 3). All eight pigs received 1 L of isotonic glucose immediately after arrival to our laboratory facility because of their overnight fast (Fig. 1). No significant differences were observed in baseline glucose and insulin levels between the two groups. Hence, the temporal variations cannot be explained by the isotonic glucose infusion. Instead, it is likely that several other mechanisms accounted for these variations. First, some studies suggest that lactate may exceed glucose as an oxidative substrate in the presence of elevated circulating lactate levels [9, 14]. The following reduction in glucose oxidation could lead to a higher blood glucose concentration. Second, an increase in blood glucose may be caused by an increased rate of gluconeogenesis following elevated plasma lactate [17, 45]. The insulin rise may be a result of the increased glucose levels, stimulating pancreatic insulin secretion. Also, lactate can increase glucagon-like peptide-1 levels [46], which can lead to a rise in insulin levels, proving another possible contributing factor.

### Clinical application

In the present study, we observed that infusion of lactate increased CO and HR while afterload was decreased. The present study was conducted in healthy pigs, with no chronic or acute illness. Hence, clinical application perspectives should be done with care. Nevertheless, some of the observations in the present study could be beneficial in situations with lowered CO and hampered metabolic flexibility i.e. heart failure. Acute and chronic HF patients may present with a low CO and an increased afterload accompanied by peripheral hypoperfusion. In acute and chronic HF, LV unloading is beneficial [47]. The present study demonstrated that lactate infusion accommodated this by reducing afterload, LVESP and P_max_ and thereby increased CO and organ perfusion without compromising MAP or increasing cardiac oxygen consumption as expressed by PVA. In contrast, we found an increase in cardiac efficiency and a decrease in the A-CS oxygen gradient across the heart. However, lactate infusion also increased HR which could be undesirable and increase all-cause mortality in HF patients with reduced ejection fraction [48]. In conjunction, this suggests that exogenous lactate may be a new potential treatment drug to unload and potentially fuel the failing heart in some situations. Larger randomised trials are warranted to investigate the effects of lactate infusions during of acute and chronic HF.

## Limitations

First, translating findings from animal research into clinical practice requires caution. To enhance clinical relevance, we chose human-sized pigs as experimental animals. Though this study only used female pigs due to the relative ease of placing a urinary catheter, pigs have close anatomical similarities with the human thoracic anatomy. Also, their cardiovascular and respiratory physiology and biochemical parameters closely resemble those of humans [49]. We chose a non-surgical, minimally invasive closed chest pressure–volume loop model rather than a traditional open chest model. Open chest cardiac catheterisation may lead to changes in key haemodynamic variables such as MAP, mPAP, CO, LVEF and afterload [33, 50]. In consideration of ethical concerns and adherence to the principles of the 3R framework, we deliberately opted for a smaller sample size to minimize the use of animals in our study. To enhance the efficiency of treatment comparisons, a crossover design was selected over a parallel design. Nevertheless, it is important to acknowledge that the limited sample size introduces the possibility of encountering type II errors, and this limitation should be considered when interpreting the study results.

We observed carryover effects of the alkalising lactate effect in the group receiving lactate as the first infusion, despite the washout period. Although alkalosis per se may improve myocardial function [51], we saw a rapid decrease in CO after termination of lactate infusion in the group receiving lactate as the first infusion (Fig. 3), even though the pigs were still highly alkalotic at this point (Additional file 1: Fig. S4). Also, P(v-a)CO_2_ could be sensitive to pH alterations. However, P(v-a)CO_2_ quickly returned to baseline levels after termination of lactate infusion in the group receiving lactate as the first infusion while pH levels were still increased (Additional file 1: Figs. S2 and S4). Therefore, it is unlikely that the greater cardiovascular effect during lactate infusion was mediated by alkalosis. Even in studies comparing hypertonic sodium lactate with hypertonic sodium HCO_3_^−^ during septicaemia, sodium lactate has superior beneficial haemodynamic effects, including stabilising effects on CO, SvO_2_ and MAP [20, 21]. Thus, it appeared unlikely that a vasodilatory effect of increased HCO_3_^−^ levels should be responsible for the greater CO during lactate infusion in the present study.

Hypertonicity and osmolality alterations may lead to plasma expansion and increased preload, increased cardiac contractility and SVR reduction, ultimately increasing CO [30–32]. Furthermore, hypernatremia per se may have a positive inotropic effect [52]. Even though other studies found lactate infusions to have more favourable haemodynamic effects than hypertonic control infusions [20, 29, 32] we aimed to keep results independent of sodium load and any associated extracellular volume expansion. Therefore, we used an iso-osmolar and -volemic hypertonic control infusion with the same sodium load as the lactate infusion. Because of the identical sodium loads, it is unlikely that hypernatremia can explain the haemodynamic differences during lactate and control infusions. Despite the presence of hypertonicity and hypernatremia, we found significant haemodynamic effects during lactate infusion including increased CO.

Cardiac efficiency is calculated using SW and PVA. Whereas SW and PVA were not significantly altered, cardiac efficiency was significantly increased during lactate infusion. Caution should always be taken when interpreting a single statistically significant parameter. However, cardiac efficiency has been shown to be increased with higher lactate levels in human patients with HF [39] and in rat hearts after haemorrhagic shock [24, 40]. Also, lactate has a higher ATP yield per oxygen molecule than FFAs [41, 42]. Furthermore, myocardial lactate consumption increases with elevated levels of circulating lactate [14]. Hence, we believe that the results in the current study remain reliable. Still, the results should be seen as hypothesis generating and further studies confirming clinical application of lactate infusions are warranted.

We catheterised CS to evaluate changes in A-CS difference of metabolites across the heart. Though a pig model is suitable for cardiovascular physiology studies and displays close similarities to the human thoracic anatomy, the cardiac vein and CS functional anatomy vary from those of their human counterparts [34]. We controlled for correct positioning using fluoroscopy and contrast injection and through serial measurements of saturation in blood samples. The calculated A-CS differences have limitations as coronary blood flow was not measured. Hence, the observed decrease in oxygen-difference across the heart during lactate infusion could be the result of increased CO and associated relatively less oxygen uptake.

In a previous study, higher levels of the ketone body 3-OHB were reported during lactate infusion [23] and 3-OHB has been demonstrated to enhance ventricular systolic function and increase CO [6, 53–55]. As we found no difference in 3-OHB concentrations, this cannot explain the haemodynamic findings.

## Conclusions

Lactate infusion increased CO by 2.0 L/min compared with control infusion. The increase in CO was caused by increase in heart rate and reduction of systemic vascular resistance and arterial elastance. No change was observed in preload measures. Furthermore, lactate infusion improved peripheral perfusion and cardiac efficiency.

### Supplementary Information

**Additional file 1: Figure S1.** Cardiac output for each animal. Cardiac output for each animal, n = 8. The four animals in the lactate to control group (**A**) and the four animals in the control to lactate group (**B**) plotted from baseline, T0, to study end at T300. Each animal is shown with connected black lines. The pink line marks the mean of the group. The grey masked area from T120 to T180 marks the washout period**. Figure S2.** Haemodynamic values. Haemodynamic values plotted from baseline, T0, to study end at T300. The grey masked area from T120 to T180 marks the washout period. Data are expressed as mean ± standard deviation (SD). Each group comprised four animals. *HR* heart rate, *MAP* mean arterial pressure, *mPAP* mean pulmonary artery pressure, *RAP* right atrial pressure, *PAWP* pulmonary artery wedge pressure, *SvO2*=mixed venous saturation, *P(v-a)CO2* venoarterial CO2 difference, *SVR* systemic vascular resistance. **Figure S3.** Schematic pressure-volume loops during lactate infusion compared with control infusion. Schematics of representative pressure-volume loop during lactate (black) compared with placebo (pink). The drawings were made using mean values at the end of each infusion period. **Figure S4.** Biochemical parameters. Temporal evolution of arterial pH, bicarbonate concentration, sodium concentration and apparent strong ion difference from baseline to study end. The grey area from T120 to T180 marks the washout period. Data are expressed as mean ± standard deviation (SD).

## Data Availability

The data supporting the conclusions of this study are available from the corresponding authors upon reasonable request.
