# Supplementary material for: Lactate infusion elevates cardiac output through increased heart rate and decreased vascular resistance: a randomised, blinded, crossover trial in a healthy porcine model
Source: J Transl Med. 2024 Mar 16;22:285. doi: 10.1186/s12967-024-05064-3 (PMC10943846; doi:10.1186/s12967-024-05064-3)
Supplement: Supplementary file 1 — Additional file 1: Figure S1. Cardiac output for each animal. Cardiac output for each animal, n = 8. The four animals in the lactate to control group (A) and the four animals in the control to lactate group (B) plotted from baseline, T0, to study end at T300. Each animal is shown with connected black lines. The pink line marks the mean of the group. The grey masked area from T120 to T180 marks the washout period. Figure S2. Haemodynamic values. Haemodynamic values plotted from baseline, T0, to study end at T300. The grey masked area from T120 to T180 marks the washout period. Data are expressed as mean ± standard deviation (SD). Each group comprised four animals. HR heart rate, MAP mean arterial pressure, mPAP mean pulmonary artery pressure, RAP right atrial pressure, PAWP pulmonary artery wedge pressure, SvO2=mixed venous saturation, P(v-a)CO2 venoarterial CO2 difference, SVR systemic vascular resistance. Figure S3. Schematic pressure-volume loops during lactate infusion compared with control infusion. Schematics of representative pressure-volume loop during lactate (black) compared with placebo (pink). The drawings were made using mean values at the end of each infusion period. Figure S4. Biochemical parameters. Temporal evolution of arterial pH, bicarbonate concentration, sodium concentration and apparent strong ion difference from baseline to study end. The grey area from T120 to T180 marks the washout period. Data are expressed as mean ± standard deviation (SD). [file 12967_2024_5064_MOESM1_ESM.pdf]

Lactate infusion elevates cardiac output through increased heart rate and decreased vascular resistance – a randomised, blinded, crossover trial in a healthy porcine model

Oskar Kjærgaard Hørsdal[a,b], MS

Niels Moeslund[c] MD, PhD

Kristoffer Berg-Hansen[a,b], MD

Roni Nielsen[a,b], MD, PhD

Niels Møller[b,d], MD, PhD, DMSc

Hans Eiskjær[a,b], MD, DMSc

Henrik Wiggers[a,b], MD, PhD, DMSc

Nigopan Gopalasingam[a,b], MD

[a]Department of Cardiology, Aarhus University Hospital, Denmark
[b]Department of Clinical Medicine, Aarhus University, Denmark
[c]Department of Heart, Lung, and Vascular Surgery, Aarhus University Hospital, Denmark
[d]Department of Endocrinology and Metabolism, Aarhus University Hospital, Denmark

Additional file 1

Page 2: Detailed animal ethics

Page 3-6: Supplementary Figures

Animal ethics

One week prior to conducting the study, the pigs arrived at the farm to acclimatise and were evaluated for signs of distress or suffering several times by professional staff. No immune or genetic modifications were used, and the animals followed the Danish Specific Pathogen Free Program. The pigs were fed a standard diet, had free access to water and were housed two-by-two in concrete floor pens.

Additional file 1: Figure S1: Cardiac output for each animal

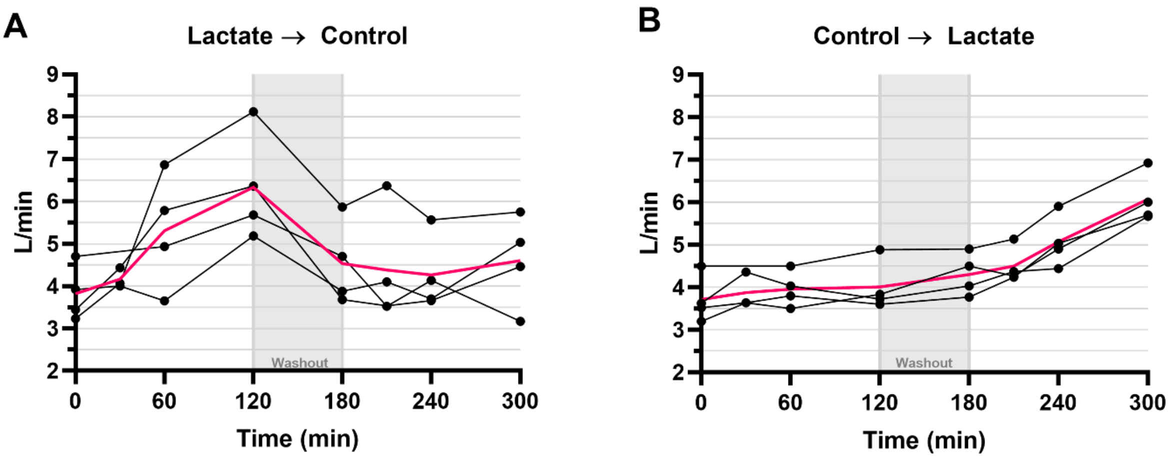

Additional file 1: Figure S1: Cardiac output for each animal, n=8. The four animals in the lactate to control group (A) and the four animals in the control to lactate group (B) plotted from baseline, T0, to study end at T300. Each animal is shown with connected black lines. The pink line marks the mean of the group. The grey masked area from T120 to T180 marks the washout period.

Additional file 1: Figure S2: Haemodynamic values

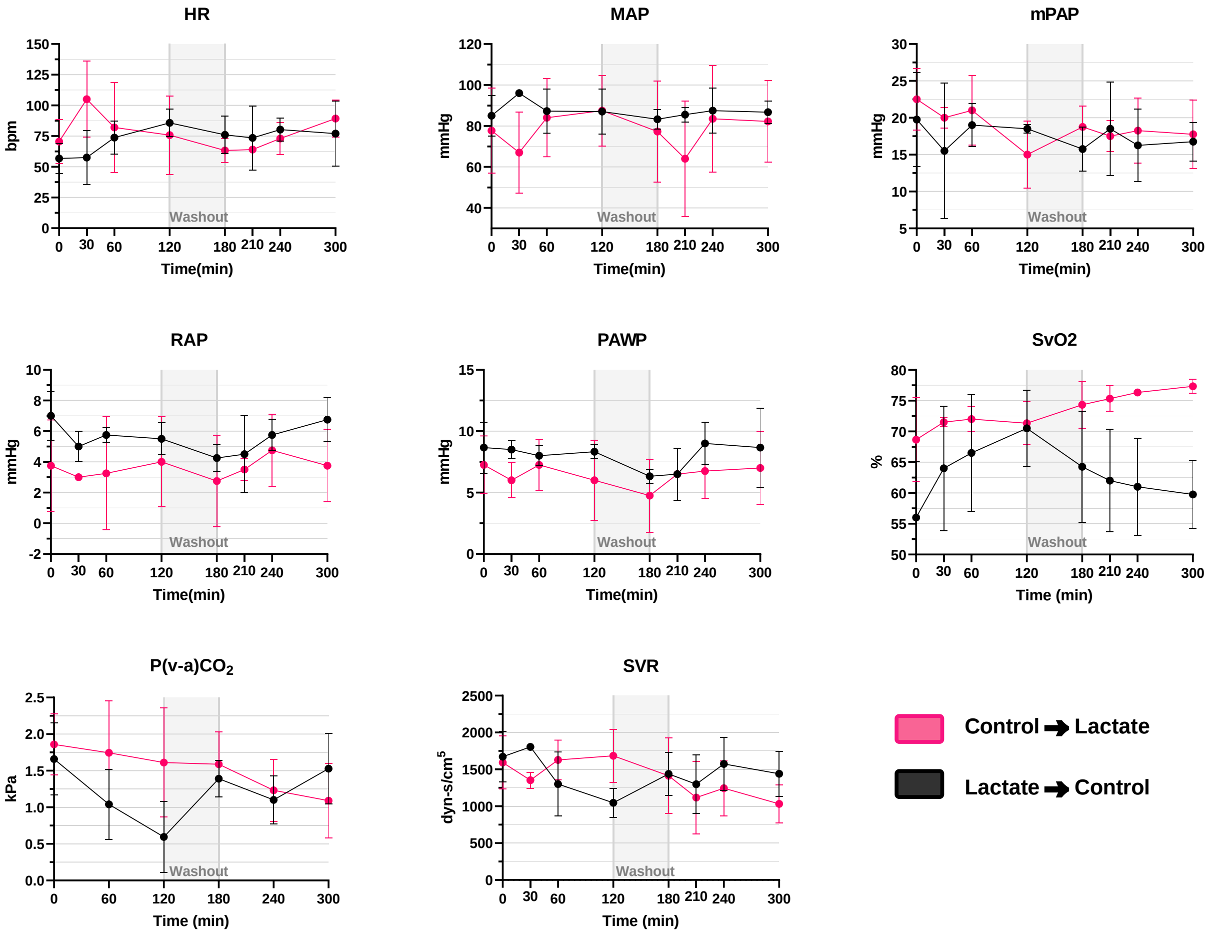

Additional file 1: Figure S2: Haemodynamic values plotted from baseline, T0, to study end at T300. The grey masked area from T120 to T180 marks the washout period. Data are expressed as mean ± standard deviation (SD). Each group comprised four animals. HR=heart rate, MAP=mean arterial pressure, mPAP=mean pulmonary artery pressure, RAP=right atrial pressure, PAWP=pulmonary artery wedge pressure, SvO2=mixed venous saturation, P(v-a)CO2=venoarterial CO2 difference, SVR=systemic vascular resistance.

Additional file 1: Figure S3: Schematic pressure-volume loops during lactate infusion compared with control infusion

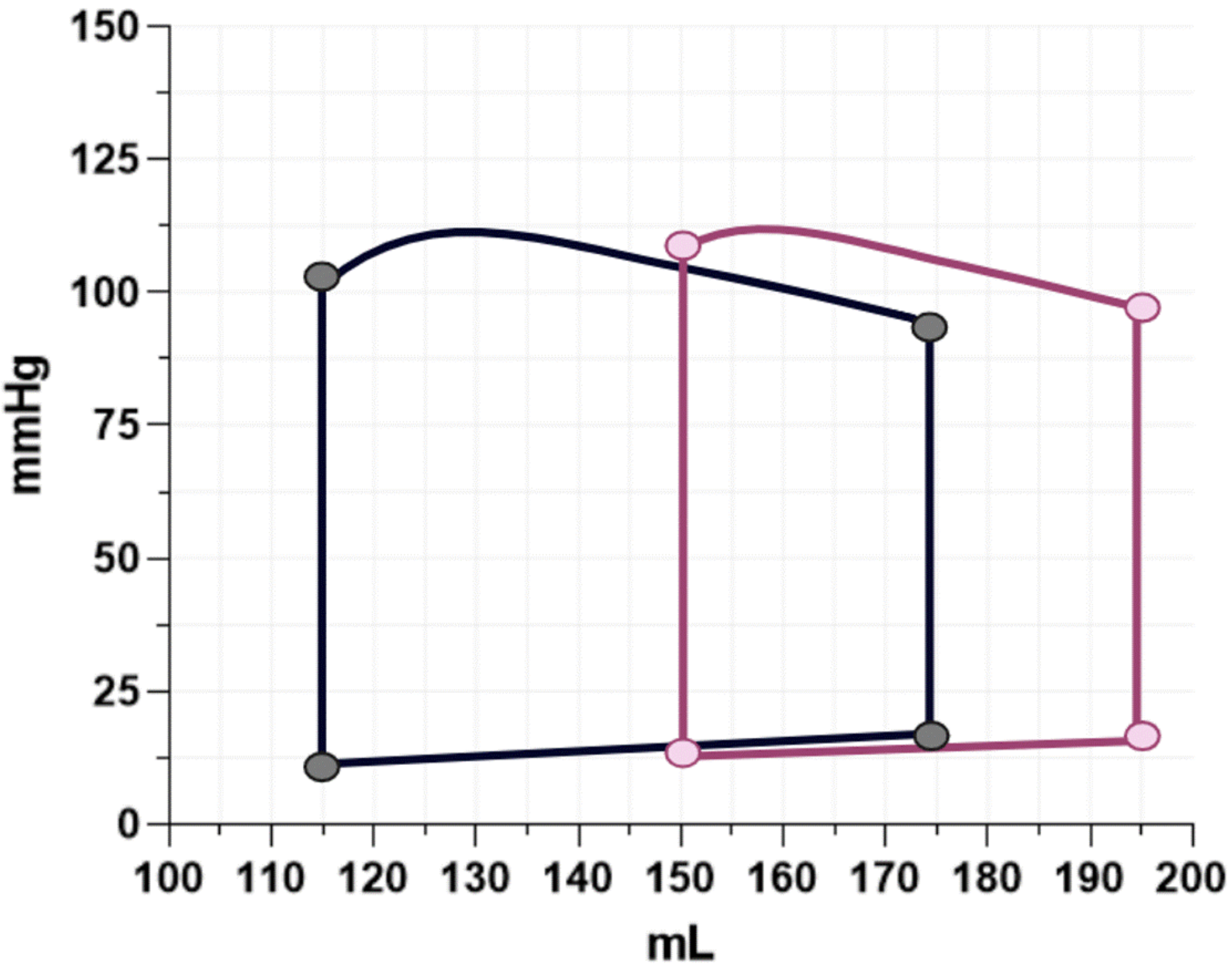

Additional file 1: Figure S3: Schematics of representative pressure-volume loop during lactate (black) compared with placebo (pink). The drawings were made using mean values at the end of each infusion period.

Additional file 1: Figure S4: Biochemical parameters

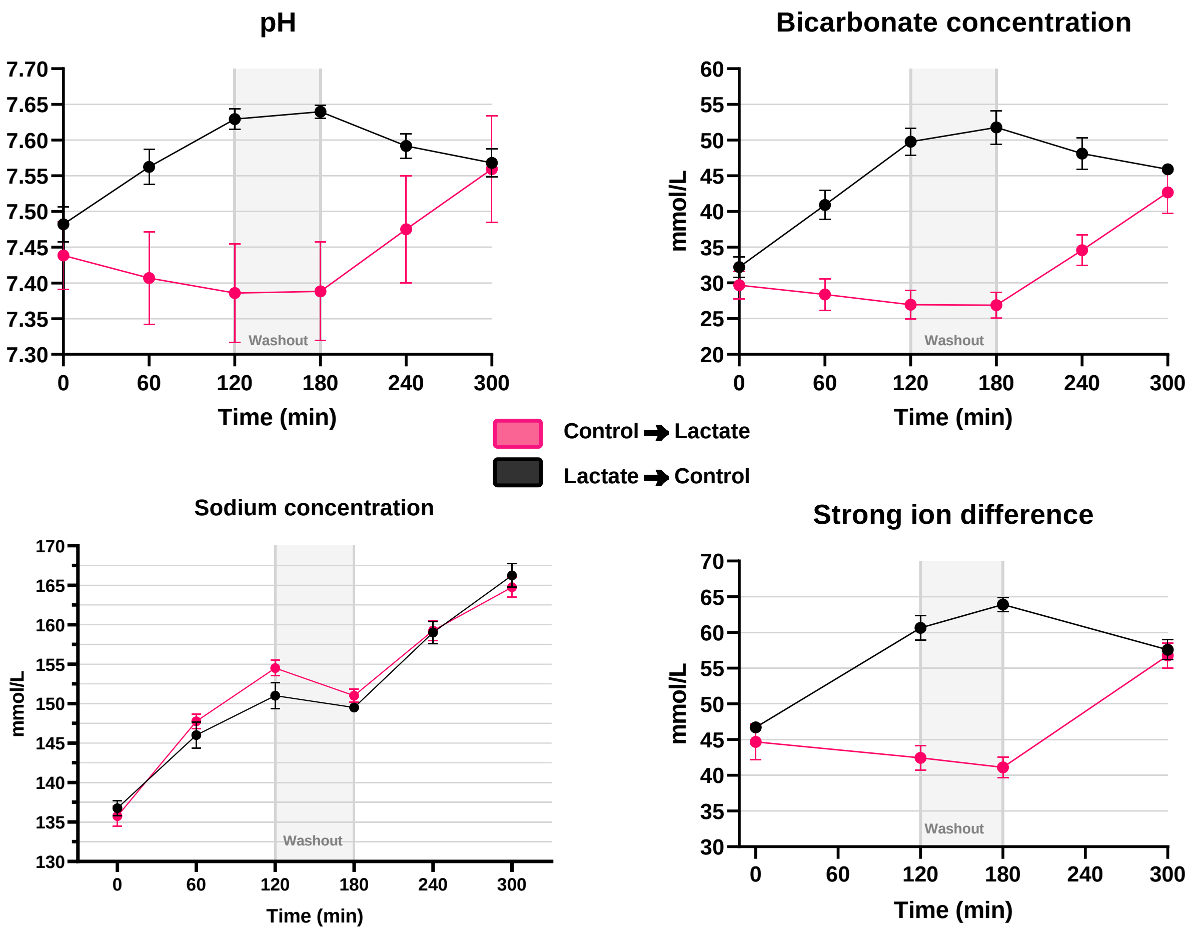

Additional file 1: Figure S4: Temporal evolution of arterial pH, bicarbonate concentration, sodium concentration and apparent strong ion difference from baseline to study end. The grey area from T120 to T180 marks the washout period. Data are expressed as mean ± standard deviation (SD).
